# Clinico-Immunological Status and Neurocognitive Function of Perinatally Acquired HIV-Positive Children on cART: A Cross-Sectional Correlational Study in South Africa

**DOI:** 10.3389/fneur.2020.00243

**Published:** 2020-04-17

**Authors:** Antonio G. Lentoor

**Affiliations:** Department of Clinical Psychology, School of Medicine, Sefako Makgatho Health Sciences University, Pretoria, South Africa

**Keywords:** neurocognitive deficits, perinatally acquired HIV (PHIV), combination antiretroviral therapy (cART), plasma CD4^+^ lymphocyte count, neurological deficits, immunological status, neurocognitive assessment

## Abstract

Despite the undisputed benefits of combination antiretroviral therapy (cART), perinatally acquired human immunodeficiency virus (PHIV) children on treatment often present with a spectrum of neurological deficits known as HIV-associated neurocognitive impairment. Even higher CD4 cell count does not seem to prevent the development of neurocognitive impairment in children with PHIV. While CD4 cell count has shown to have the greatest prognostic value, its association with neurocognitive abilities remains to be clarified. This study aimed at determining the correlation between plasma CD4^+^ lymphocyte and neurocognitive function in children with PHIV on cART. In total, 152 purposively recruited hospital-based sample of children with PHIV on cART, aged 3 years to 7 years 6 months (mean age, 63.13 months), underwent neurocognitive assessment using the Wechsler Preschool and Primary Scale of Intelligence, Third Edition. Immunological status of each child was based on the plasma CD4^+^ lymphocyte levels. The mean CD4^+^ lymphocyte cell count at the time of neurocognitive assessment was 1,259.85 cells/mm^3^ (mean range, 139–2,717 cells/mm^3^), with significant age difference on CD4^+^ lymphocyte count levels [*F*_(2, 149)_ = 13.58, *p* = 0.000]. CD4^+^ lymphocyte counts was significantly correlated with subdomains of neurocognitive function scores of task that measures working memory, processing speed, and perceptual reasoning. Global cognitive ability (Full Scale Intellectual Quotient) had no significant association with immunological status of the children. The findings support an association between immunological status of PHIV infection and executive function task. These neurocognitive faculties are critical for learning, school readiness and success in early childhood, and ultimately treatment adherence in adolescence. The need for early identification of neurodevelopment deficits in children, even when on cART, is crucial because early psychosocial and neurorehabilitative interventions can lead to better outcome for children with PHIV.

## Introduction

While progress has been made toward the UNAIDS 90-90-90 targets for prevention and treatment, human immunodeficiency virus (HIV) infection continues to be a major global public health issue (1). According to the Global AIDS update 2019 report, the vast majority of people living with HIV is in low- and middle-income countries, with an estimated 66% living in sub-Saharan Africa, while South Africa accounts for a third of all new HIV infections in this region (2). While HIV prevalence remains high in the general population in South Africa, the report indicated a rapid decline in new infections among South African children, from 25,000 in 2010 to 13,000 in 2017. It is reported that of the estimated 280,000 children (aged 0–14 years) living in South Africa, 58% were on treatment (3). This is mainly due to South Africa's antiretroviral treatment program, which is considered to be the largest in the world and most successful in sub-Saharan Africa. Moreover, dramatic expansion of South Africa's antiretroviral therapy (ART) program over the years has led to everyone with a positive diagnosis being eligible to start and access to treatment (4).

Combination ART (cART) has emerged as the most effective drug regimen to maximally suppress and stop the progression of HIV disease and is considered the standard of care for the treatment of pediatric HIV (5). Human immunodeficiency virus damages the immune system because it targets CD4^+^ T-lymphocyte cells. As an infected CD4^+^ cell multiplies, fewer HIV-free working CD4^+^ cells become available, eventually decreasing the concentration of these cells in the blood (CD4^+^ count) (6). Consequently, patients become susceptible to a wide range of opportunistic infections, especially if they are not receiving cART.

It goes without saying that advances made in antiretroviral treatment have transformed the disease into a chronic but manageable illness that enabled children to live longer with HIV. Although early initiation of cART has resulted in great improvements in survival, neurological consequences remain an important comorbidity among children living with HIV (7). The neuropathogenesis of HIV-associated neurocognitive disorder in the era of cART is complex and among others includes persistent systematic and central nervous system inflammation, oxidative stress, and HIV-subtype (clade) neurovirulence variation, associated with neuronal death and potential neurotoxicity of cART drugs (8). Some research reported that children with perinatally acquired HIV (PHIV) present more frequently than adults with central nervous system opportunistic infection resulting from HIV immunosuppression due to the vulnerability of the child's developing brain (9). Neurological manifestations in perinatally HIV infected children include cortical and subcortical structural changes (10) that is associated with neurocognitive impairments in PHIV children and is argued to contribute to the overall burden of neurodevelopmental disabilities that persist throughout childhood, adolescence, and beyond (11). Domain-specific neurocognitive impairments have been found in children with HIV even on cART (12). Yet, there is still a limited understanding of the exact degree of these neurocognitive impairments in PHIV children. They have been found to perform poorly of tests that measure particularly the domain of executive functioning, notably processing speed, working memory, planning and attention, visuospatial ability, and visual memory and planning (13, 14).

Although marked immune compromise, as evidenced by low CD4^+^ lymphocyte counts, is now less frequently found among children with access to cART, milder forms of neurocognitive impairments are still prevalent. Yet the association between the CD4^+^ lymphocyte count and neurocognitive function in children on cART remains unclear. This study, therefore, aims to address this gap and contribute to the understanding of the domain-specific neurocognitive function of PHIV children on cART.

## Materials and Methods

### Participants

A cross-sectional quantitative study comprising 152 child–caregiver dyads was conducted between January 2012 and December 2013 in a hospital-based purposive sample from a periurban community on the outskirts of East London, Eastern Cape, South Africa. Generally, most people are living under very poor conditions with high unemployment rates and food insecurity in this community, and the tertiary hospital serves as a referral center for the broader Eastern Cape catchment areas.

The primary caregivers of children aged between 3 and 7 years 6 months with perinatal HIV infection on cART, at the time of the study, were invited during a routine weekly pediatric HIV outpatient clinic to participate in the study. Combination ART was defined as a regimen of three or more antiretroviral drugs, typically from two drug classes (Table 1). Besides the routine follow-up clinical and physical examination by the pediatrician, children of primary caregivers who consented to participate in the study underwent neurocognitive assessment on the Wechsler Preschool and Primary Scale of Intelligence, Third Edition (WPPSI-III). Also, CD4^+^ lymphocyte measurements were taken by the pediatrician for each child (Table 2).

**Table 1:** Summary of recommended first-line ART regimens for children and adolescents.

	**Children 3 to <10 year and adolescents <35 kg**	**Adolescents (10–19 year) ≥35 kg**
Preferred	ABC + 3TC + EFV	TDF + 3TC (or FTC) + EFV
Alternatives	ABC + 3TC + NVP	AZT + 3TC + EFV
	AZT + 3TC + EFV	AZT + 3TC + NVP
	AZT + 3TC + NVP	TDF + 3TC (or FTC) + NVP
	TDF + 3TC (or FTC) + EFV	
	TDF + 3TC (or FTC) + NVP	
Special	d4T + 3TC + EFV	ABC + 3TC + EFV
circumstances	d4T + 3TC + NVP	ABC + 3TC + NVP

*These recommendations apply to children and adolescents who initiate first-line ART as per WHO Clinical Guidelines*.

**Table 2:** Sociodemographic, clinicoimmunological, and cognitive characteristics of the HIV-positive children (*N* = 152) in the study.

**Age of children (months)**			
Mean age	63.13		
Age range	31.38–92.78		
**Gender**	**n (%)**		
Boys	65 (42.8)		
Girls	87 (57.2)		
**Child's level of education**			
Not attending	46 (30.3)		
Crèche	13 (8.6)		
Grade R	46 (30.3)		
Primary	47 (30.9)		
**CDC Staging**			
1	81		
2	15		
3	4		
Mean CD4 count (cells/mm^3^)	1,259.85		
Mean CD4 count range	139–2,717		
**CD4 count ≤5 years**		**WHO Grade Immunosuppression**	
≥1,000	38 (69.1)	Asymptomatic	*p*
500–999	15 (27.3)	Mild to moderate	0.000
≤ 500	2 (3.6)	Severe	
**CD4 count ≥6 years**			*p*
≥500	62 (89.9)	Asymptomatic	0.000
200–499	4 (5.8)	Mild to moderate	
≤ 200	3 (4.3)	Severe	
**Cognitive function**	**Boys**	**Girls**	***t***
FSIQ	78.98 ± 12.91	83.33 ± 12.48	2.09^*^
VIQ	76.51 ± 10.49	78.52 ± 11.75	1.09
PIQ	86.08 ± 15.61	92.23 ± 17.56	2.24

* *Significance level p < 0.01. t = statistical test. FSIQ, Full Scale Intellectual Quotient; VIQ, Verbal Intellectual Quotient; PIQ, Performance Intellectual Quotient*.

### Measures

#### Immunological Status

##### CD4+ Lymphocytes levels

A meta-analysis of untreated HIV infected children from resource-limited setting showed that CD4^+^ lymphocyte count has the greatest prognostic value and so was used here (15). The CD4^+^ grading was done according to the World Health Organization revised classification of immunosuppression in children into stage 1, stage 2, and stage 3 based on CD4^+^ lymphocyte counts of >1,000 cells/mm^3^, 500–999 cells/mm^3^, <500 cells/mm^3^, respectively (16). All participants received the first-line cART consisting of lopinavir/ritonavir (LPV/r)–based regimen in combination with two nucleoside/nucleoside reverse transcriptase inhibitors, including zidovudine, lamivudine/emtricitabine, abacavir, and efavirenz. Studies have shown LPV/r-based regimens to have a better safety profile and to be more effective in HIV-infected children (17, 18).

### Neurocognitive Function

An assessment tool that was able to assess a breadth of neurocognitive skills appropriate for school-aged children and has fewer language demands was needed. The WPPSI-III was selected for the study because it one of the most widely used and tested scales of neurocognitive functioning for preschool and school-aged children. The assessment tool was developed for children of 2½ to 7 years 3 months. Evidence of adequate validity and reliability in a number of low- and middle-income countries, such as Bangladesh, Philippines, Jamaica, Indonesia, and Brazil, has been established (19). The WPPSI-III subtest has also been used to test neurocognitive functioning in HIV research with children from low-income backgrounds and representative of the larger population of children living with HIV (20). The assessment consists of seven subtests that determine the following domains of neurocognitive function: verbal, performance, working memory, and processing speed that combined generate an estimate of general cognitive ability. The reference mean for general neurocognitive ability [Full Scale Intellectual Quotient (FSIQ)] and each subtest is 100, with a standard deviation of 15, whereas subtest-scaled scores have a mean of 10 and standard deviation of 3. The assessments were conducted in accordance with the procedure in manual in the morning to lessen the impact of diurnal variation and fatigue in performance.

### Data Analysis

The Shapiro–Wilk normality test was conducted to test the assumptions of normality, whereas the Levene test was used to observe the assumptions of equality and homogeneity of variance in the variables. The correlation between CD4^+^ lymphocyte cell count and neurocognitive function was determined using the Pearson correlation. Group comparisons between age of children, gender of children, and category of CD4^+^ lymphocyte levels in relation to the children's neurocognitive function were assessed using one-way analysis of variance test (ANOVA) and independent-sample *t*-test. All data analysis was conducted with the statistical package IBM SPSS (21.0) (IBM Incorporation, Chicago, IL, USA). All tests conducted were two-tailed and held at a statistical significance of *p* < 0.05 with the confidence interval set at 95% level.

## Results

The mean general neurocognitive ability score was subnormal (81.47 ± 12.81). Neurocognitive function was significantly associated with age and gender of the children, with boys performing worse (78.98 ± 12.91) than girls (83.33 ± 12.48) in the study (*t* = 2.09, *p* < 0.05). Older boys performed worse than older and younger girls in the study [*F*_(2,149)_ = 14.42, *p* < 0.001].

Vertical transmission was the only mode of transmission in the study group. Of 152 children on cART, more than 90% had their CD4^+^ count ≥500 cells/mm^3^, were asymptomatic, and were considered immunologically competent. Other 4.8% of children were mild to moderately immunosuppressed with CD4^+^ count in between 200 and 499 cells/mm^3^, whereas only 2.4% were severely immunosuppressed with CD4^+^ count of <200 cells/mm^3^. The mean CD4^+^ lymphocyte cell count at the time of neurocognitive assessment was 1,259.85 cells/mm^3^ (mean range, 139–2,717 cells/mm^3^). There was a significant age difference on CD4^+^ count levels [*F*_(2,149)_ = 13.58, *p* = 0.000], with older age children (≥6 years) evidencing higher CD4^+^ count levels than those of younger children.

CD4^+^ lymphocyte counts were significantly correlated with subdomains of neurocognitive function scores, but not with global neurocognitive ability (FSIQ). Significant correlations were observed for CD4^+^ count with scores on the neurocognitive tasks of block design (*r* = 0.19, *p* = 0.033), symbol search (*r* = 0.22, *p* = 0.036), and word reasoning (*r* = 0.22, *p* = 0.021), picture completion (*r* = 0.23, *p* = 0.026), and comprehension (*r* = 0.20, *p* = 0.049) (Table 3). These are complex neurocognitive tasks that involve the frontal cortex and parietal lobe. No significant differences were observed on neurocognitive function across the different CD4^+^ level categories (Table 4).

**Table 3:** Correlations among CD4^+^ lymphocyte levels cells/mm^3^ and subdomains of cognitive function in the study of HIV-positive children.

**Variables**	**Mean (SD)**	**CD4^**+**^ count**	**Pic**	**Comp**	**WR**	**BD**	**SS**
CD4^+^ count	1,259.85 (565.8)	–	0.230*	0.204*	0.218*	0.197*	0.217*
Pic	3.79 (2.42)	–	–	0.663**	0.658**	0.103	0.180
Comp	5.75 (2.66)	–	–	–	0.676**	0.163	0.409**
WR	6.28 (2.29)	–	–	–	–	0.146	0.282**
BD	5.92 (3.46)	–	–	–	–	–	−0.059
SS	2.52 (1.47)	–	–	–	–	–	–

Correlation is significant at *p <0.05 (two-tailed), **p <0.01 (two-tailed). CD4 count, CD4^+^ lymphocyte levels cells/mm^3^; Pic, picture completion; Comp, comprehension; WR, word reading; BD, block design; SS, symbol search.

**Table 4:** One-way ANOVA results for CD4^+^ lymphocyte levels cells/mm^3^, subdomains, and composite cognitive function in the study of HIV-positive children.

	**CD4**^****+****^ **count**								
**Cognitive function**	**≤200 cells/mm**^**3**^		**Between 200 and 499 cells/mm**^**3**^		**≥500 cells/mm**^**3**^		**95% Confidence interval for mean**		
**Subdomain test**	**Mean**	**SD**	**Mean**	**SD**	**Mean**	**SD**	**df**	***F***	***p***
Comprehension	3.00	1.00	3.20	0.84	3.21	0.77	1	0.09	0.77
Symbol search	1.33	0.58	1.60	0.89	1.99	0.76	1	3.03	0.85
Word reading	3.00	1.00	3.40	0.55	3.42	0.74	1	0.38	0.54
Picture completion	4.00	0.00	3.80	0.45	4.36	1.23	1	1.23	0.27
Block design	2.33	1.15	3.00	1.26	3.15	1.08	1	0.97	0.33
**Composite test**									
Full scale intellectual function	74.00	9.165	79.67	6.346	82.32	13.017	2	0.727	0.49
Verbal intellectual function	73.00	5.292	78.67	5.922	77.83	11.901	2	0.272	0.76
Performance intellectual function	82.00	5.196	86.17	6.824	90.62	17.792	2	0.531	0.58

## Discussion

In this study, the influence of CD4^+^ lymphocyte cell count on neurocognitive performance in HIV-positive children on cART was evaluated. Consistent with previous studies, the findings from the present study showed that perinatally HIV-infected children are at high risk of developing neurocognitive impairment, even when on cART (21, 22). Consistent with the findings of a study conducted among HIV-infected children on treatment in India, neurocognitive impairment was observed despite being on ART treatment for more than 2 years with an undetectable viral load and normal CD4^+^ cell count (23). Similar findings were found in a South African study that reported HIV-infected children with a median age of 5 years did not show improvement in neurocognitive function after 6 months of ART (24). In addition, the finding from the present study is consistent with studies showing that children infected with HIV relative to uninfected groups evidence focal neurocognitive deficits on task that measures working memory (25); processing speed (26); visual motor integration, sustained attention (27); and motor speed coordination (28). Unlike in the present study, these studies did not find impairment on global intellectual functioning. This difference, however, can be attributed to issues related to study designs, clade types, treatment status, disease stage, and neurocognitive assessment tools used (29).

Although researchers have reached varying conclusions, the finding from this study is in agreement with the studies that found a correlation between neurocognitive performance and CD4^+^ lymphocyte count among asymptomatic HIV-positive children on cART. Levels of CD4^+^ lymphocyte have been found to not only provide useful information concerning the progression to AIDS and death but also identify those individuals at risk of neurocognitive impairment before more severe opportunistic infections occur (30).

The majority in this age group (33.9%) had CD4^+^ levels >500 cells/mm^3^. For both age groups, those with a higher CD4^+^ count were associated with better performances on focal neurocognitive task that measures working memory, motor, and processing speed. This finding is consistent with several studies that reported on neurocognitive effects after a relatively short period of treatment. For the older age group, it reflects perhaps the influence of longer period of cART on neurocognitive development (31). This finding underscores the importance of early and sufficient cART, even if the neurocognitive benefits are modest.

Similar to the present findings, Boccellari et al. (32) found a significant relationship between CD4^+^ count and cognition when comparing individuals with CD4^+^ count of <200 cells/mm^3^ with CD4^+^ count >400 or >500 cells/mm^3^. However, this was not the case when comparing the current findings to Ghate et al. (33) that found no difference in the overall intelligence quotient scores with a stratified CD4^+^ count (<350 and >350 cells/mm^3^) in their sample of HIV-positive children in India. The reason for this difference in finding might be due to the majority of patients in the Indian study having had a low mean CD4^+^ cell count (588 cells/mm^3^) in comparison to the current sample (mean CD4^+^ cell count 1,259.85 cells/mm^3^). In addition, the difference in finding might be due to HIV-1 clade C isolates that exist in sub-Saharan Africa, which has been found to be genetically distinct from those in Southern Asia and to contribute to differential neuropathogenic properties (34). Of the various subtypes of HIV-1 clade C, which predominates in sub-Saharan Africa (including South Africa) and Southeast Asia (including India), any functional change in HIV-1 clade C isolate may have a significant impact on the neurovirulence of the infection (35). It has been reported that HIV-1 clade C found in Africa has an important natural variation in the dicysteine motif C30C31 of the Tat protein that promotes viral replication directly and plays a pivotal role in neurotoxicity. Variants with the dicysteine motif in Tat are one of the key viral determinants of neurocognitive dysfunction in patients with HIV-1 clade C (36). As such, higher incidence of neuropathogenic affects observed in Africa as opposed to Southern Asia has been associated with higher frequency of variants of dicysteine motif in Tat in HIV-1 clade C, whereas the reduced neurocognitive dysfunction incidence observed in Southern Asia, in countries such as India, is due to loss of Tat monocyte chemotactic property (37). This difference is an important observation for neurological dysfunction associated with HIV-1 clade C infection of the developing central nervous system, especially of children in Southern Africa (38). Neuronal apoptosis induced by the HIV-1 clade C Tat protein has been demonstrated in the brain of patients *in vivo* and has been associated with dementia in the adult population (39). This has important implications for treatment strategies to prevent neuronal death and improve on neurological outcomes in people living with HIV. More so, given that all the children in this study were on cART, with a higher average CD4^+^ cell count associated with higher neurocognitive scores, but still perform in the subnormal intellectual range across domains of neurocognitive functioning, an important question is raised around the potential neuroprotective value of early treatment (40), while keeping in mind the complex nature of the developing brain of children. Furthermore, cART's restorative role on neurocognitive function is another important point of consideration, especially because findings have been varied when it comes to drug penetrance in the central nervous system, which has been shown to be a reservoir for the virus (41).

The study had some limitations. The use of a cross-sectional design precludes drawing any causal relationship between CD4^+^ lymphocyte count and neurocognitive function in PHIV-positive children. Although the cohort comprised 152 PHIV-positive children, it still had insufficient power that limits the potential to generalize the findings. Because no neurotypical and/or socioeconomically matched children cohorts were used to control for comorbidities and environmental factors, it was not possible to conduct a control group analysis that could have strengthened the results. In addition, the durations of cART treatment before assessment varied, and this could lead to bias. There is also a possibility of selection bias, as very sick children were not enrolled into this cohort. However, this cohort of PHIV-positive children all attended the tertiary hospital for care and may therefore represent the most affected group of children. Despite these limitations, the study has drawn attention to an important area of research that needs further exploration in the era of cART.

## Conclusion

Neurocognitive vulnerability in perinatally HIV infected children underscores the need for early recognition of these deficits and emphasizes the importance of not only early and effective antiretroviral treatment (42) but also the need for timeous neurocognitive rehabilitative interventions (43), more so because HIV affects the brain during a very crucial time of central nervous system development in perinatally infected children. Efforts to improve timely access to pediatric HIV diagnosis and cART (44), including psychosocial interventions, should therefore remain a global public health priority. Further, the findings from this study point to the need for larger longitudinal studies to better understand the effects of HIV on the neuropathogenesis of HIV-associated neurocognitive impairment throughout the brain development process under standard cART treatment (38).

## Data Availability Statement

The datasets generated for this study are available on request to the corresponding author.

## Ethics Statement

The studies involving human participants were reviewed and approved by Biomedical Research Ethics Committee (Protocol Number: BE252/11) of the University of KwaZulu-Natal and from the Institutional Review Board of the hospital. Written informed consent to participate in this study was provided by the participants' legal guardian/next of kin. The study adhered to the Ethical principles of the Declaration of Helsinki (45). Caregivers were informed of the availability of counseling and social-welfare services should the need for these arise.

## Author Contributions

The author confirms being the sole contributor of this work and has approved it for publication.

### Conflict of Interest

The author declares that the research was conducted in the absence of any commercial or financial relationships that could be construed as a potential conflict of interest.
